# Inhibiting ALK-TOPK signaling pathway promotes cell apoptosis of ALK-positive NSCLC

**DOI:** 10.1038/s41419-022-05260-3

**Published:** 2022-09-27

**Authors:** Juanjuan Xiao, Lu Zhang, Huijun Yi, Ling Zou, Jianmei Mo, Feng Xue, Jinhua Zheng, Yingze Huang, Hui Lu, Hansheng Wu, Peipei Xue, Xin Zhang, Lifei He, Zhaoxin Li, Shigui Pang, Guibin Qiao, Qiuhong Duan, Feng Zhu

**Affiliations:** 1 Cancer Research Institute, The Affiliated Hospital of Guilin Medical University, Guilin, 541001 Guangxi People’s Republic of China; 2 Guangxi Health Commission Key Laboratory of Novel Onco-Kinases in Target Therapy, The Affiliated Hospital of Guilin Medical University, 541001 Guilin, Guangxi People’s Republic of China; 3 Department of Biochemistry and Molecular Biology, School of Basic Medicine, Huazhong University of Science and Technology, 430030 Wuhan, Hubei People’s Republic of China; 4 The Fifth Clinical Medical College of Henan University of Chinese Medicine (Zhengzhou People’s Hospital), 450053 Zhengzhou, Henan People’s Republic of China; 5 Department of Oncology, The Affiliated Hospital of Guilin Medical University, 541001 Guilin, Guangxi People’s Republic of China; 6 Department of Pathology, The Affiliated Hospital of Guilin Medical University, 541001 Guilin, Guangxi People’s Republic of China; 7 Wuhan Children’s Hospital (Wuhan Maternal and Child Healthcare Hospital), Tongji Medical College, Huazhong University of Science and Technology, 430015 Wuhan, Hubei People’s Republic of China; 8 Department of Thoracic Surgery, The first affiliated hospital of Shanton University Medical College, 515041 Shantou, Guangdong People’s Republic of China; 9 Department of Thoracic Surgery, Guangdong Provincial People’s Hospital, Guangdong Academy of Medical Sciences, 510080 Guangzhou, People’s Republic of China; 10 The Second School of Clinical Medicine, Southern Medical University, 510515 Guangzhou, People’s Republic of China; 11 NHC Key Laboratory of Birth Defects and Reproductive Health, Chongqing Population and Family Planning Science and Technology Research Institute, 400000 Chongqing, People’s Republic of China; 12 Guangxi Key Laboratory of Molecular Medicine in Liver Injury and Repair, the Affiliated Hospital of Guilin Medical University, 541001 Guilin, Guangxi People’s Republic of China; 13 Present Address: Guangxi Health Commission Key Laboratory of Novel Onco-Kinases in Target Therapy, The Affiliated Hospital of Guilin Medical University, 541001 Guilin, Guangxi People’s Republic of China

**Keywords:** Targeted therapies, Growth factor signalling

## Abstract

T-LAK cell-oriented protein kinase (TOPK) is a potential therapeutic target in tumors. However, its role in anaplastic lymphoma kinase (ALK)-positive non-small cell lung cancer (NSCLC) has not been reported. Here, we found that TOPK was highly expressed in ALK-positive NSCLC. Additionally, ALK was identified as another upstream kinase of TOPK by in vitro kinase assay screening. Then, it was proven that ALK phosphorylated TOPK at Y74 in vitro and ex vivo, and the pathways downstream of ALK-TOPK were explored by phosphoproteomic analysis. Subsequently, we demonstrated that inhibiting TOPK enhanced tumor sensitivity to alectinib (an ALK inhibitor). The combination of alectinib and HI-032 (a TOPK inhibitor) suppressed the growth and promoted the apoptosis of ALK-positive NSCLC cells ex vivo and in vivo. Our findings reveal a novel ALK-TOPK signaling pathway in ALK-positive NSCLC. The combination of alectinib and HI-032 might be a promising therapeutic strategy for improving the sensitivity of ALK-positive NSCLC to targeted therapy.

## Introduction

Lung cancer is the leading cause of cancer-related deaths worldwide, and an estimated 1.8 million individuals died of lung cancer in 2020 [1]. NSCLC accounts for more than 80% of lung cancer cases. ALK-driven tumors develop in ~3–8% of patients with NSCLC, and development of these tumors is associated with important patient features: more than 90% of these patients have never smoked or had only light smoking habits, were female, younger at diagnosis, where diagnosed with adenocarcinoma by histology, and lacked other oncogenic drivers [2, 3]. The fusion of the echinoderm microtubule-associated protein-like 4 (EML4) gene with ALK gene is the most common ALK rearrangement in NSCLC.

The rearranged EML4-ALK is constructively activated through oligomerization, which is mediated by EML4, and the fusion product interacts with a variety of downstream kinases or connector molecules to activate downstream signaling pathways that lead to tumorigenesis [4]. It has been reported that constructive ALK can inhibit cell death and promote cell proliferation and tumorigenesis via canonical signaling pathways, including the JAK/STAT3, RAS-RAF-MEK-ERK, PLCγ and phosphoinositide 3-kinase (PI3K)-AKT-mTOR pathways [5]. In addition, active ALK can phosphorylate SMAD4 at Tyr95, disable the DNA-binding activity of SMAD4 and then abrogate TGF-β-mediated tumor suppression responses [6]. Thus, ALK inhibitors such as crizotinib, ceritinib, alectinib and loratinib have been developed and used in clinical treatment. ALK-positive NSCLC patients have a high rate of response to these inhibitors, and these inhibitors can achieve a better clinical outcome and longer progression-free survival than other therapies; however, similar to other TKIs, most patients also develop drug resistance to ALK inhibitors [7]. Therefore, it is necessary to explore the mechanism underlying the development of ALK-positive NSCLC, search for new therapeutic targets, and further optimize targeted therapy.

TOPK, which is also named PBK (PDZ-binding kinase), has increasingly been considered to be a specific target for cancer therapy [8]. TOPK is highly expressed in a variety of neoplastic tissues, but it is almost not expressed or expressed at low levels in normal tissues [9]. TOPK can directly phosphorylate histone H3 [10], ERK2 [11], c-Jun [12], H2AX [13], Prx-1 [14] and PRPK [15], thus promoting tumor growth, metastasis and drug resistance. Moreover, it has been reported that OTS964 and OTS514, which are TOPK inhibitors, effectively inhibit cell proliferation [16]. Previously, we found that Src and MET were upstream kinases of TOPK, and the phosphorylation of TOPK by Src and MET promoted the tumorigenesis of colon cancer and resistance of NSCLC to gefitinib, respectively [17, 18]. Moreover, other upstream kinases of TOPK also attracted our attention.

Here, ALK was identified as another upstream kinase of TOPK by in vitro kinase assay screening. Subsequently, we found that ALK phosphorylated TOPK at Y74, and inhibiting both ALK and TOPK enhanced the apoptosis of ALK-positive NSCLC cells ex vivo and in vivo. Our findings might offer a novel strategy for the treatment of ALK-positive NSCLC.

## Materials and methods

### Cell culture and transfection

H2228, H1975, H1299, and HEK293T cells were purchased from American Type Culture Collection (ATCC; Manassas, VA). Cell lines were authenticated by periodic short tandem repeat (STR) profiling and confirmed mycoplasma negative status before being frozen. All cell lines were thawed and passaged at least three times before initiating experiments. H2228, H1975 and H1299 cells were grown in RPMI-1640 medium (Gibco, Grand Island, NY) supplemented with 10% fetal bovine serum (FBS; Gibco, Grand Island, NY), and HEK293T cells were cultured in Dulbecco’s modified Eagle’s medium (DMEM, Gibco, Grand Island, NY) supplemented with 10% FBS. The cells were cultured at 37 °C in a humidified 5% CO_2_ atmosphere. Cell transfection assays were performed using Simple-fect (Signaling Dawn Biotech, Wuhan, HB) following the manufacturer’s instructions. The corresponding blank vectors were added to ensure that the total amount of DNA transfected in each group was equal. Then, we used puromycin (Sigma, St. Louis, MO) to screen cell lines in which gene expression was stably silenced.

### Plasmids and shRNAs

Flag-EML4-ALK(V3A) and Flag-EML4-ALK(V3B) were kindly provided by Prof. Hiroyuki Mano (Tokyo University, Bunkyo-ku, TKY). HA-TOPK, pMD2.0G, and psPAX2 were purchased from Addgene (Cambridge, MA). The plasmids of phage-Flag-EML4-ALK(V3A), phage-Flag-EML4-ALK(V3B), pLKO.1-shALK (#1-#4), pcDNA3-HA-TOPK(Y74F), pet46-His-TOPK(Y74F) were constructed by our laboratory. Additionally, the lentivirus plasmids shALK (#1, forward oligo, 5’ - CCGGTCAGATGATAGCCGTAATAAACTCGAGTTTATTACGGCTATCATCTGATTTTTG -3’; #2, forward oligo, 5’-CCGGCTCTTCCTTGGGATCCCTAAGCTCGAGCTTAGGGATCCCAAGGAAGAGTTTTTG -3’; #3, forward oligo, 5’-CCGGCTTCGCTGACTGCCAATATGACTCGAGTCATATTGGCAGTCAGCGAAGTTTTTG -3’; #4, forward oligo, 5’-CCGGTGTGCCATGCTGCCAGTTAAGCTCGAGCTTAACTGGCAGCATGGCACATTTTT-3’), shTOPK (#1, forward oligo, 5’- CCGGGGGAACTAGGCCACCTATTAACTCGAGTTAATAGGTGGCCTAGTTCCCTTTTTG -3’; #2, forward oligo, 5’-CCGGGAAGTGTGGCTTGCGTAAATACTCGAGTATTTACGCAAGCCACACTTCTTTTTG-3’); #3, forward oligo,5’-CCGGGTAATGATCATTATCGAAGTGCTCGAGCACTTCGATAATGATCATTACTTTTTG-3’; #4, forward oligo, 5’-CCGGCCTTCAGAAGGTTGCTGAGTACTCGAGTACTCAGCAACCTTCTGAAGGTTTTTG-3’; #4, forward oligo, 5’-CCGGGCCTTCATCATCCAAACATTGCTCGAGCAATGTTTGGATGATGAAGGCTTTTTG-3’; #5, forward oligo, 5’-CCGGGATTCCACACATTAATCTTTCCTCGAGGAAAGATTAATGTGTGGAATCTTTTTG-3’) were obtained from Sangon Biotech, Inc. The pLKO.1-puro Non-Target shRNA Control Plasmid DNA (shMock) was purchased from Sigma-Aldrich. All constructs were confirmed by restriction enzyme mapping, DNA sequencing, and the Blast program.

### Antibodies and reagents

TOPK (sc-293028) was purchased from Santa Cruz Technology, Inc (Santa Cruz, CA). Flag (F1804) was purchased from Sigma-Aldrich (St. Louis, MO). Antibodies to detect ALK (#3633), p-ALK (#3341), p-JNK (#4668), JNK (#9258), p-ATF2 (#5112), ATF2 (#9226), PARP (#9542), c-PARP (#5625), HA (#3724), p-Tyrosine (#8954) were purchased from Cell Signaling Technology (Danvers, MA). β-actin (66009-1-Ig),α-Tubulin (66031-1-Ig), HRP-labeled Goat anti-Mouse IgG (H + L) and Goat anti-Rabbit IgG (H + L) were from Proteintech Group, Inc (Wuhan, HB). Phospho-TOPK at Y74 antibody was prepared by Abgent, Inc (Suzhou, JS). All antibodies were used following the instructions of the respective manufacturers.

### Anchorage-independent cell transformation assay

For this experiment, cells (8 × 10^3^ cells/well) were seeded in 6-well plates and then cultured with 2× BME (Sigma, St. Louis, MO), 200 mM l-glutamine, 50 mg/ml gentamicin, 1.25% agar (Sigma, St. Louis, MO) and 10% FBS agar mix. The cells were cultured at 37 °C in a humidified 5% CO_2_ atmosphere for 14 days, and then, the numbers of colonies that formed were counted and scored using Image-Pro Plus software.

### MTT assay and growth curve analysis

Cells (7 × 10^3^ cells/well) were seeded in 96-well plates and incubated for 24 h. Then, the cells were exposed to different concentrations of TOPK inhibitor (HI-032) or ALK inhibitor (alectinib) for 72 h. Subsequently, 100 μl of 0.5% MTT was added to each well and incubated for 4 h. Then, the reaction was terminated with 150 μl of dimethyl sulfoxide (DMSO). Finally, GraphPad Prism software was used to analyze cell viability according to the absorbance measured at 492 nm with an enzyme immunoassay analyzer (Tecan Infinite 200 PRO, Switzerland). For growth curve analysis, cells were plated at 6 × 10^4^ cells per 6 cm dish, and cells numbers were counted in triplicate at different time points using a hemocytometer to generate a growth curve.

### In vitro kinase assay

ALK, EGFR, FAK, FLT-3, Fyn, JAK2, Src, VEGFR2 active kinase and ATP were purchased from Millipore Corporation (Billerica, MA). Kinase buffer (10×) was purchased from Cell Signaling Technology (Danvers, MA). His-TOPK (WT) and His-TOPK (Y74F) were expressed in *Escherichia coli* BL21 bacteria. The peptides were synthesized by GL Biochem Ltd. (Shanghai, China). The inactive substrate (3 μg) and the active kinase (1 μg) were incubated at 37 °C for 2 h in 1× kinase buffer supplemented with 100 μM ATP or 1 μCi [γ-32P]-ATP (China Isotope & Radiation Corporation, Beijing, China). The samples were added to 5× loading buffer and then analyzed by autoradiography or western blotting.

### In vivo assay

Female NOD-Prkdc^scid^ Il2γg^null^ (NPG) mice aged 6–8 weeks (18–20 g, *n* = 40) were purchased from WeiTongDa Company (Beijing, China). H2228 cells (5 × 10^6^ cells/0.1 ml RPMI-1640 medium, 0.1 ml Matrigel) were subcutaneously inoculated into the right flank of each mouse. After 10 days, 24 mouse tumors successfully grew to an average of 300 mm^3^ each. They were randomly divided into four groups (*n* = 6 per group) and given the drug trial. Vehicle control or alectinib (2 mg/kg) was orally, HI-032 (10 mg/kg) was administered by intraperitoneal injection, or the two treatments were given in combination. All the groups were continuously treated for three weeks. Double blinding was performed in this experiment. The mice were weighed every 2 days during this period, and tumor volume was calculated using the ellipsoid formula (length × width × height × 0.52). The animal maintenance and experimental procedures were approved by the Animal Care Committee of Wuhan Servicebio Technology Co., Ltd. (Wuhan, China).

### Western blotting assay

Cells were cultured for western blotting and lysed with RIPA buffer (Beyotime Biotechnology, Shanghai). The cell lysates were sonicated by ultrasound for 2.5 s (20% power) each time, with a pause of 3.5 s for 2 min between each sonication. Then, the lysates were centrifuged at 12,000 rpm at 4 °C for 30 min, and then, the protein concentrations were measured by the Bradford method. The samples were separated using 7.5%–15% SDS–PAGE gels and transferred to polyvinylidene difluoride (PVDF) membranes (Millipore, Billerica, MA). The membranes were then blocked with TBST solution supplemented with 5% nonfat milk or 5% BSA for 30 min and incubated with the primary antibody at 4 °C overnight, followed by incubation with the appropriate second antibody. The results were then visualized an imaging system (Bio-Rad, USA) using chemiluminescence. All of our full and uncropped western blots were shown in the supplemental material (Supplementary S1).

### Pull-down analysis

Cells were cultured for western blotting, lysed with IP buffer (50 mM Tris-HCl pH 7.4, 150 mM NaCl, 1 mM EDTA, 1 mM DTT, and 1% NP40), then homogenized with 1 ml syringes and centrifuged at 12,000 rpm at 4 °C for 30 min. A small amount of supernatant was retained as a positive control. The remaining supernatants were divided into two tubes, and 20 μl NTA-His-Control beads (Santa Cruz, CA, USA) and 20 μl prokaryotes expressing NI-NTA-His-TOPK (WT) beads were added. The beads were placed in 4 °C refrigerators and mixed for 48 h. These samples were washed with IP buffer three times, and a western blotting assay was performed.

### Flow cytometry analysis

Cell death was measured using the Annexin V Fluorescein Isothiocyanate (FITC)/Propidium Iodide (PI) Apoptosis Assay Kit (Elabscience, Wuhan, HB). Cells (6 × 10^5^/well) were seeded in 6-well plates and cultured at 37 °C in a humidified 5% CO_2_ atmosphere for 24 h. After treatment with alectinib and/or HI-032 for 24 h, the cells were digested and centrifuged, and the precipitates were washed with precooled PBS three times. Then, the precipitates were resuspended with 1× binding buffer to a density of 2 × 10^6^/ml, and the cells were incubated for 15 min at room temperature with Annexin V-FITC plus PI according to the manufacturer’s protocol. Finally, cell death was measured by a flow cytometer (BD Biosciences, San Jose, CA).

### Analysis of the effects of the drug combination

Alectinib and HI-032 were used at different concentrations alone or in combination. Cell proliferation after 72 h of treatment was measured by MTT assay, and the effect of the combination of drugs was evaluated with the nonconstant ratio of each drug. CompuSyn (ComboSyn, Inc., Paramus, NJ) was used to assess the interaction of the combined drugs to assess synergy/additivity/antagonism using the combination index (CI) value from the Chou-Talalay method [19]. CI < 1, =1, and >1 represent synergistic, additive and antagonistic effects, respectively.

### Phosphoproteomic analysis

For the Tandem Mass Tag (TMT)-based quantitative phosphoproteome experiments, H2228 cells were infected with lentiviruses encoding control hairpin shRNA (shMock) or shRNA specific for TOPK (shTOPK#1), and the knockdown efficiency was investigated by western blotting. Then, the cells were treated with 0.1 μM alectinib for 24 h, and cell pellets were collected. Subsequently, protein samples were prepared, and liquid chromatography-tandem mass spectrometry (LC–MS/MS) was performed using the TMT technique at Jingjie PTM Biolab Co., Ltd. (Hangzhou, China). Briefly, the protein samples were digested to generate mixture of peptides, and then, the tryptic peptides were labeled with a TMT kit according to the protocol. Next, the TMT-labeled peptides were fractionated by high pH reverse-phase HPLC using an Agilent 300Extend C18 column (5 μM particles, 4.6 mm ID, 250 mm length). Then, each fraction was subjected to phosphopeptide enrichment with immobilized metal affinity chromatography (IMAC)-TiQ2. Subsequently, specific peptides were selected for in-solution digestion and LC–MS/MS, and the resulting MS data were processed using the MaxQuant search engine (v.1.5.2.8). Finally, bioinformatics methods were used to analyze the MS data.

### Patient samples and immunohistochemistry (IHC)

We collected 34 ALK-positive NSCLC specimens and 40 paracancerous tissue specimens from Guangdong Provincial People’s Hospital of China. This experiment was approved by the medical ethics committee of Guangdong Provincial People’s Hospital and informed consent was obtained from all subjects. Paraffin-embedded tissues were sectioned at 5 µm thickness. The slides were deparaffinized, rehydrated, and treated with 3% hydrogen peroxide for 25 min. Antigen retrieval was performed in citrate buffer pH 6.0 in a microwave oven for 8 min. After the slides were blocked with 3% bovine serum albumin in phosphate-buffered saline (PBS) for 30 min, the tissue sections were incubated overnight at 4 °C with the indicated primary antibodies. PBS and mouse or rabbit IgG1 (Santa Cruz Biotechnology, CA) were used as the blank and negative controls, respectively. The sections were imaged using an Olympus Imaging System Microscope (BX51, Olympus, Tky). The immunostaining intensity was graded following the Remmele scoring method. Samples with a score > 3 were included in the positive group, and the others were included in the negative group.

### Statistical analysis

All the experiments were repeated three times, and the results shown are representative of the results of the three experiments. Statistical analysis was conducted using the *t*-test. *P < 0.05, **P < 0.01, ***P < 0.001, and ns indicates no significant difference.

## Results

### ALK phosphorylates TOPK at Y74 in vitro

**Fig. 1:**
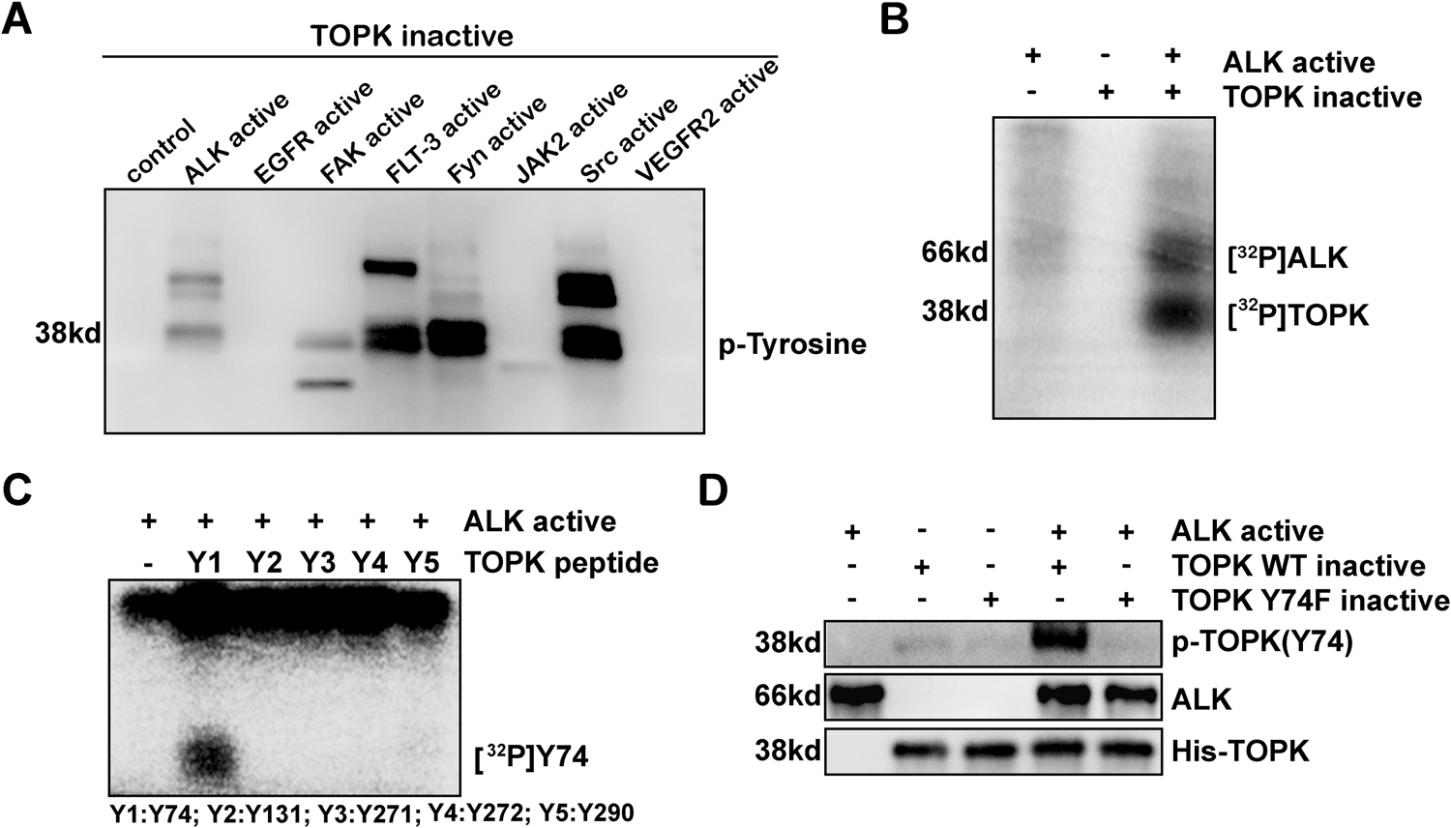
ALK phosphorylates TOPK at Y74 in vitro. **A** Screening for upstream kinases of TOPK. P-Tyrosine was detected by western blotting analysis. **B** Active ALK phosphorylated inactive TOPK in vitro in the presence of [γ-32p] ATP as visualized by autoradiography. **C** Synthesized peptides containing potential tyrosine sites were used as substrates in an in vitro kinase assay with active ALK in the presence of [γ-32P] ATP and the results were visualized by autoradiography. **D** Active ALK phosphorylated inactive TOPK-WT or 74F in vitro in the presence of ATP. Then, the samples were analyzed by western blotting. Data are representative of results from triplicate experiments.

In our previous studies, TOPK was shown to be phosphorylated by Src [17] and MET [18] and to be a promising target for cancer therapy. Whether TOPK could be regulated by other kinases attracted our attention. Therefore, some active kinases, such as ALK, EGFR, FAK, FLT-3, Fyn, JAK2, Src and VEGFR2, and the inactive form of TOPK were used to perform in vitro kinase assays. The results suggested that ALK, FAK, FLT-3 and Fyn might also phosphorylate TOPK, similar to Src (Fig. 1A and Supplementary S1). Next, ALK was chosen for further study because it is closely associated with NSCLC. The active form of ALK was incubated with the inactive form of TOPK in the presence of [γ-^32^P] ATP, and an in vitro kinase assay was conducted. The data indicated that ALK could phosphorylate TOPK (Fig. 1B). To further identify the specific phosphorylation sites, five peptides (Y74, Y131, Y271, Y272, and Y290) that had been previously selected and synthesized [17] were individually subjected to an in vitro kinase assay with the active form of ALK. The autoradiography results showed that the Y74 peptide of TOPK was markedly phosphorylated by ALK (Fig. 1C). Subsequently, the wild-type TOPK (WT) protein and a TOPK protein in which the Y74 site was mutated and rendered inactive (TOPK (74F)) were purified from *E. coli* and used as substrates for the active form of ALK in another in vitro kinase assay. Western blotting analysis using the specific p-TOPK (Y74) antibody confirmed that ALK could phosphorylate TOPK at the Y74 site (Fig. 1D and Supplementary S1). These results demonstrate that ALK can phosphorylate TOPK at Y74 in vitro.

### TOPK is highly expressed in ALK-positive NSCLC

**Fig. 2:**
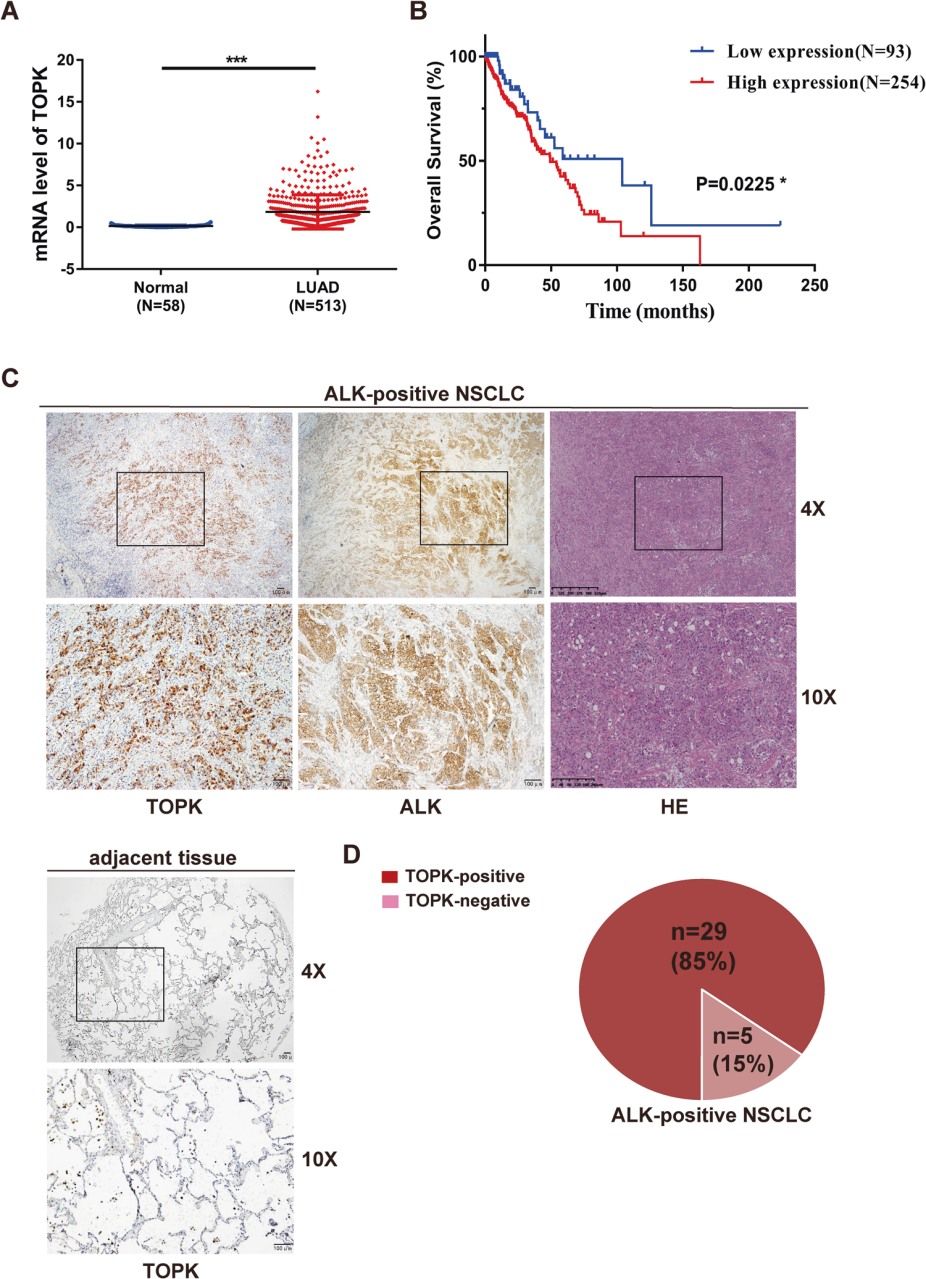
TOPK is highly expressed in ALK-positive NSCLC. **A** Differential expression analysis of TOPK on LUAD datasets of TCGA. ***P < 0.001. **B** Kaplan–Meier analysis of overall survival in LUAD patients. The data came from the online cBioPortal database. *P < 0.05. **C**, **D** IHC staining of TOPK in adjacent tissues and ALK-positive NSCLC tissue samples. Representative images were shown (**C**). Magnifications, ×40, ×100, scale bar = 100 µm.

Next, we explored the expression of TOPK in NSCLC patients. The TCGA data from cBioPortal database showed that the mRNA level of TOPK in lung adenocarcinoma (LUAD) tissues was much higher than that in normal tissues, and patients with high expression of TOPK had a lower survival rate (Fig. 2A, B). To further determine the role of TOPK in ALK-positive NSCLC, the expression of TOPK in 40 adjacent tissue specimens and 34 ALK-positive NSCLC specimens was measured by IHC staining. The results showed that the protein level of TOPK was increased in ALK-positive NSCLC tissues compared with adjacent tissues (Fig. 2C). The rate of TOPK-positive expression was as high as 85% in ALK-positive NSCLC patients (Fig. 2D). These results indicate that TOPK is highly expressed in ALK-positive NSCLC.

### ALK phosphorylates TOPK at Y74 ex vivo and enhances the stability of TOPK

**Fig. 3:**
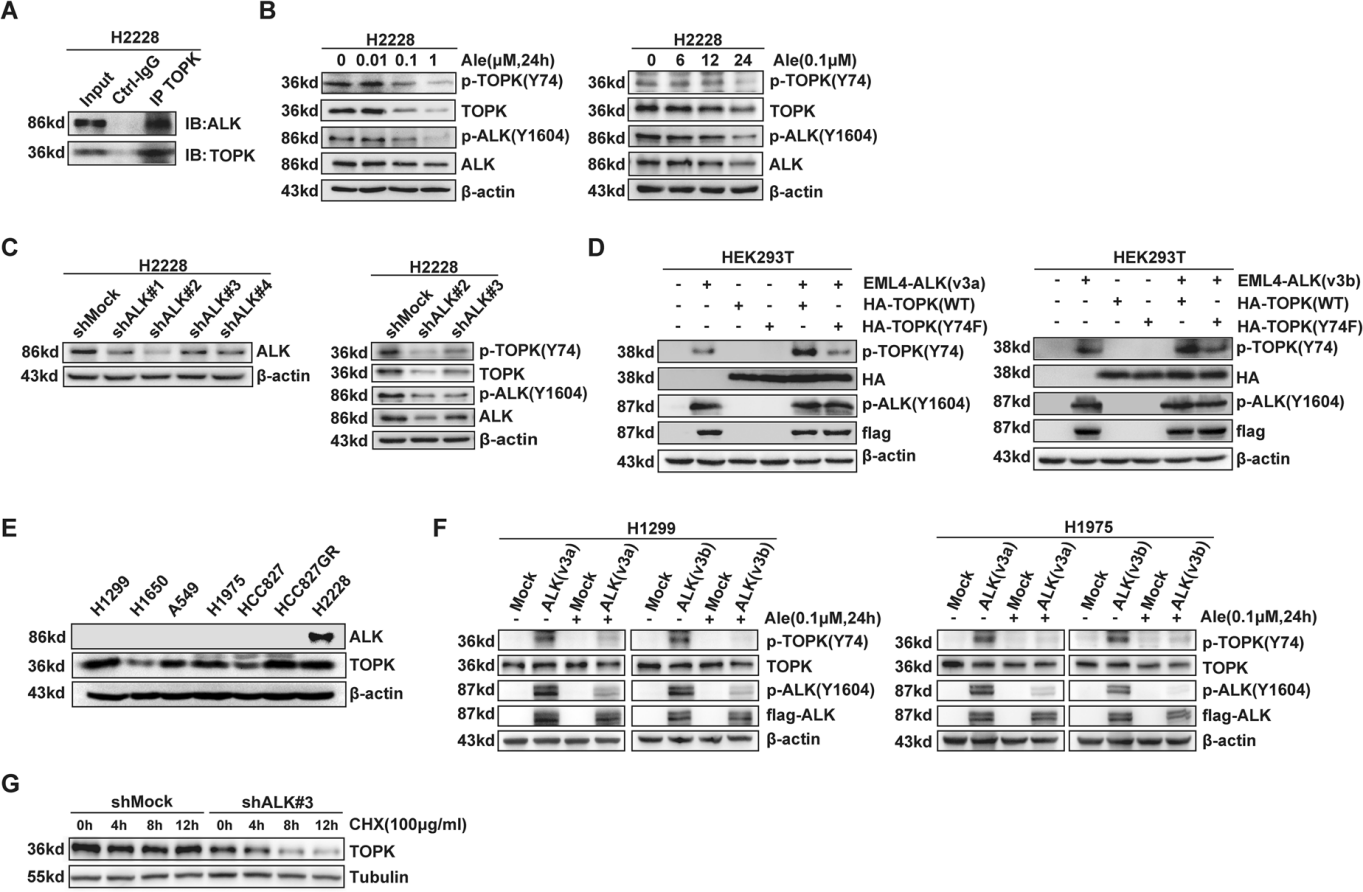
ALK phosphorylates TOPK at Y74 ex vivo and enhances the stability of TOPK. **A** TOPK bound with endogenous ALK of H2228 cells by pull-down assay. **B** H2228 cells were treated with Alectinib (0 μΜ, 0.01 μΜ, 0.1 μΜ, or 1 μΜ) for 24 h or 0.1 μM Alectinib for 0 h, 6 h, 12 h, or 24 h, and the samples were analyzed by western blotting. **C** The stable shMock and shALK in H2228 cells were established and the samples were analyzed by western blotting. **D** Flag-ALK (v3a/v3b) was cotransfected in HEK293T cells with HA-TOPK-WT or HA-TOPK 74F, and the samples were analyzed by western blotting. **E** The expression of ALK and TOPK in various lung cancer cells. **F** The stable ALK (v3a) or (v3b)-overexpression in H1299 and H1975 cells were established and the samples were analyzed by western blotting. **G** The cells were treated with CHX (100 μg/ml) for 0 h, 4 h, 8 h, and 12 h, and the samples were analyzed by western blotting. Data are representative of results from triplicate experiments.

Although it has been confirmed in vitro that ALK phosphorylates TOPK at Y74 and that TOPK is highly expressed in ALK-positive NSCLC, it is unclear whether the phosphorylation of TOPK at Y74 occurs in cells. To explore the mechanism by which TOPK is phosphorylated by ALK in cells, ALK-positive H2228 cells (express EML4-ALK variants 3a and 3b [20]) were used for subsequent experiments. First, pull-down analysis showed that ALK could bind to TOPK (Fig. 3A and Supplementary S1). Then, the endogenous level of p-TOPK (Y74) was measured in H2228 cells treated with the ALK inhibitor alectinib. The results showed that the level of p-TOPK (Y74) gradually decreased in a dose- and time-dependent manner (Fig. 3B and Supplementary S1). Subsequently, ALK-knockdown H2228 cell lines were established, and the p-TOPK (Y74) levels were measured by western blotting. The results suggested that the level of p-TOPK (Y74) was significantly reduced in the ALK-knockdown groups (Fig. 3C and Supplementary S1). Moreover, a plasmid carrying wild-type TOPK (HA-TOPK (WT)) or a plasmid carrying Y74F-mutant TOPK (HA-TOPK (74F)) was cotransfected into HEK293T cells with a EML4-ALK-v3a/EML4-ALK-v3b plasmid. The results showed that the level of p-TOPK (Y74) increased in the group cotransfected with HA-TOPK (WT) (Fig. 3D and Supplementary S1). At the same time, we tested the expression of ALK and TOPK in various lung cancer cells. Both H1299 and H1975 cells had no expression of ALK and moderate expression of TOPK (Fig. 3E and Supplementary S1). Then we set up stable EML4-ALK (v3a)/EML4-ALK (v3b)-overexpressed H1299 and H1975 cell lines. Cells were followed to be treated with 0.1 µM alectinib for 24 h and the p-TOPK (Y74) levels were measured by western blotting. The results showed that the level of p-TOPK (Y74) was greatly increased in the ALK-overexpressed groups and dramatically reduced after treatment with alectinib (Fig. 3F and Supplementary S1). Those data suggested that ALK phosphorylates TOPK at Y74 in cells. Notably, as shown in Fig. 3C, the total protein level of TOPK was obviously decreased in the ALK-knockdown groups. This result suggested that the stability of TOPK might be affected by ALK. Therefore, ALK-knockdown H2228 cells were treated with CHX to investigate the half-life of endogenous TOPK. The results indicated that the half-life of TOPK in ALK-knockdown H2228 cells was much shorter than that in control cells (Fig. 3G and Supplementary S1).

Overall, these results show that ALK phosphorylates TOPK at Y74 and enhances the stability of TOPK.

### Inhibition of TOPK attenuates the anchorage-independent growth and promotes the apoptosis of H2228 cells

**Fig. 4:**
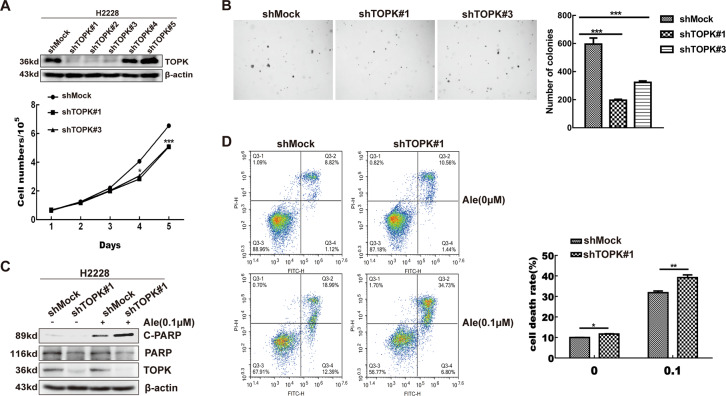
Inhibition of TOPK attenuates the anchorage-independent growth and promotes the apoptosis of H2228 cells. **A** Growth curve analysis was used to detect the proliferation rate of the stable shMock and shTOPK H2228 cells. Upper panel showed verification of the cell lines identified by western blotting. ***P < 0.001. **B** Knockdown of TOPK reduced tumorigenic properties of H2228 cells ex vivo. Representative photomicrographs of colony formation in soft agar of shMock cells compared with shTOPK#1 or shTOPK#3 cells were shown. Data are represented as mean ± standard deviation from triplicate experiments. ***P < 0.001. **C**, **D** The stable shMock and shTOPK#1 H2228 cells were treated with alectinib (0.1 μΜ) for 24 h. Total and cleaved PARP were detected by western blotting analysis (**C**). Dead cells were detected by Annexin V-FITC and PI double staining and analyzed by flow cytometry (**D**). Data are represented as mean ± standard deviation from triplicate experiments. *P < 0.05, **P < 0.01.

After verifying that ALK can enhance the stability of total TOPK, we further explored the effect of TOPK on the phenotype of ALK-positive lung cancer cells. We established stable TOPK-knockdown (shTOPK) H2228 cells, and then, the function of the cells was analyzed. The growth of the shTOPK cells was markedly slower than that of the control shMock cells (Fig. 4A and Supplementary S1). Next, the anchorage-independent colony formation of the stable cell lines was investigated. Compared with the shMock cells, the shTOPK cells formed significantly fewer and smaller colonies (Fig. 4B). Western blotting analysis showed that the cleavage of PARP (a marker of apoptosis) was notably increased in the shTOPK cells treated with 0.1 µM alectinib for 24 h (Fig. 4C and Supplementary S1). Similarly, flow cytometry revealed that knockdown of TOPK expression promoted the death of H2228 cells, especially the cells treated with 0.1 µM alectinib (Fig. 4D). These results suggest that inhibition of TOPK attenuates the anchorage-independent growth of H2228 cells and promotes apoptosis.

### Phosphoproteomic analyses of molecules downstream of TOPK

**Fig. 5:**
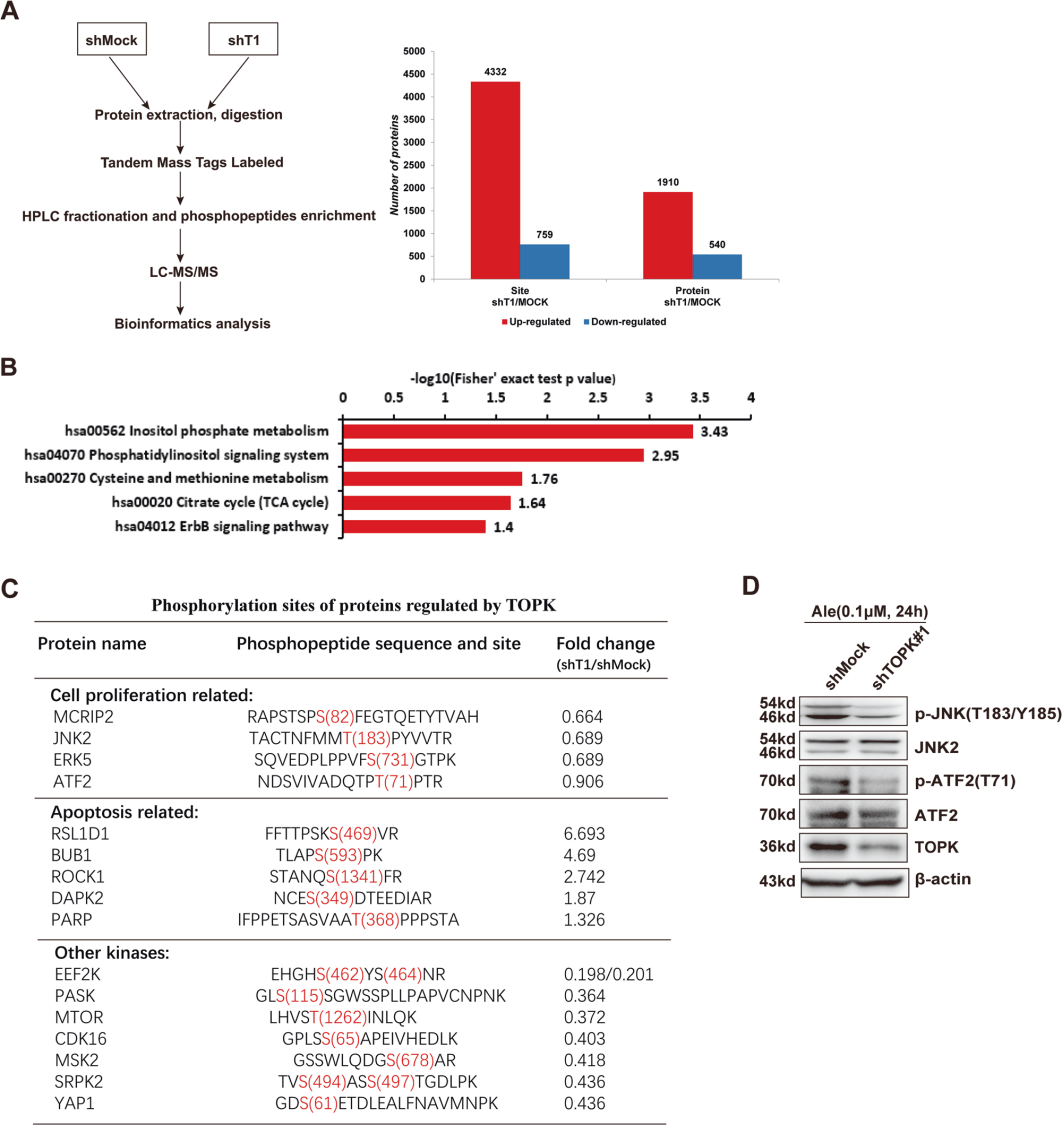
Phosphoproteomic analyses of molecules downstream of TOPK. **A** Systematic workflow of the TMT-based quantitative phosphoproteome experiments (left panel). A total of 5091 phosphosites in 2450 proteins were identified and quantified (right panel). **B** The downregulated signaling pathways in shTOPK H2228 cells. **C** A list of selected phosphoproteins regulated by TOPK were shown. **D** The expression levels of p-JNK (T183/Y185) and p-ATF2 (T71) in the shTOPK H2228 cells were analyzed by western blotting. Data are representative of results from triplicate experiments.

Based on the above studies, we further explored the mechanism downstream of the ALK-TOPK pathway. To comprehensively understand the ALK-TOPK signaling network, shMock and shTOPK#1 H2228 cells were treated with 0.1 µM alectinib for 24 h and harvested for TMT-based quantitative phosphoproteome experiments, as shown in the left panel of Fig. 5A. A total of 5091 phosphosites in 2450 proteins were identified and quantified (Fig. 5A right panel, and Supplementary S2). Enrichment-based clustering analyses were performed using the Kyoto Encyclopedia of Genes and Genomes (KEGG) database to profile cellular pathways of Q1-Q4 groups regulated by TOPK (Supplementary S3). The ErbB signaling pathway, which is related to cell proliferation, was downregulated in shTOPK H2228 cells (Fig. 5B). To provide detailed molecular information from this KEGG analysis, a list of selected phosphoproteins was shown in Fig. 5C. The phosphorylation of multiple molecules (MCRIP2, JNK2, ERK5 and ATF2) related to cell proliferation was decreased after the knockdown of TOPK expression. Some phosphorylated proteins (RSL1D1, BUB1, ROCK1, DAPK2, and PARP) that are related to apoptosis were much more highly expressed in the shTOPK cells than in the shMock cells. Potential downstream kinases whose expression levels were most significantly reduced included EEF2K, PASK, MTOR, CDK16, MSK2, SRPK2, and YAP1. In addition, we performed western blotting using existing phospho-antibodies, and the results showed that the expression levels of p-JNK (T183/Y185) and p-ATF2 (T71) were decreased in the shTOPK H2228 cells (Fig. 5D and Supplementary S1), which was consistent with the phosphoproteomic analyses. These MS data strongly support our previous results and suggest that inhibition of TOPK attenuated cell growth and promoted apoptosis.

### Treatment with a combination of alectinib and HI-032 synergistically inhibited the growth and enhanced the apoptosis of H2228 cells

**Fig. 6:**
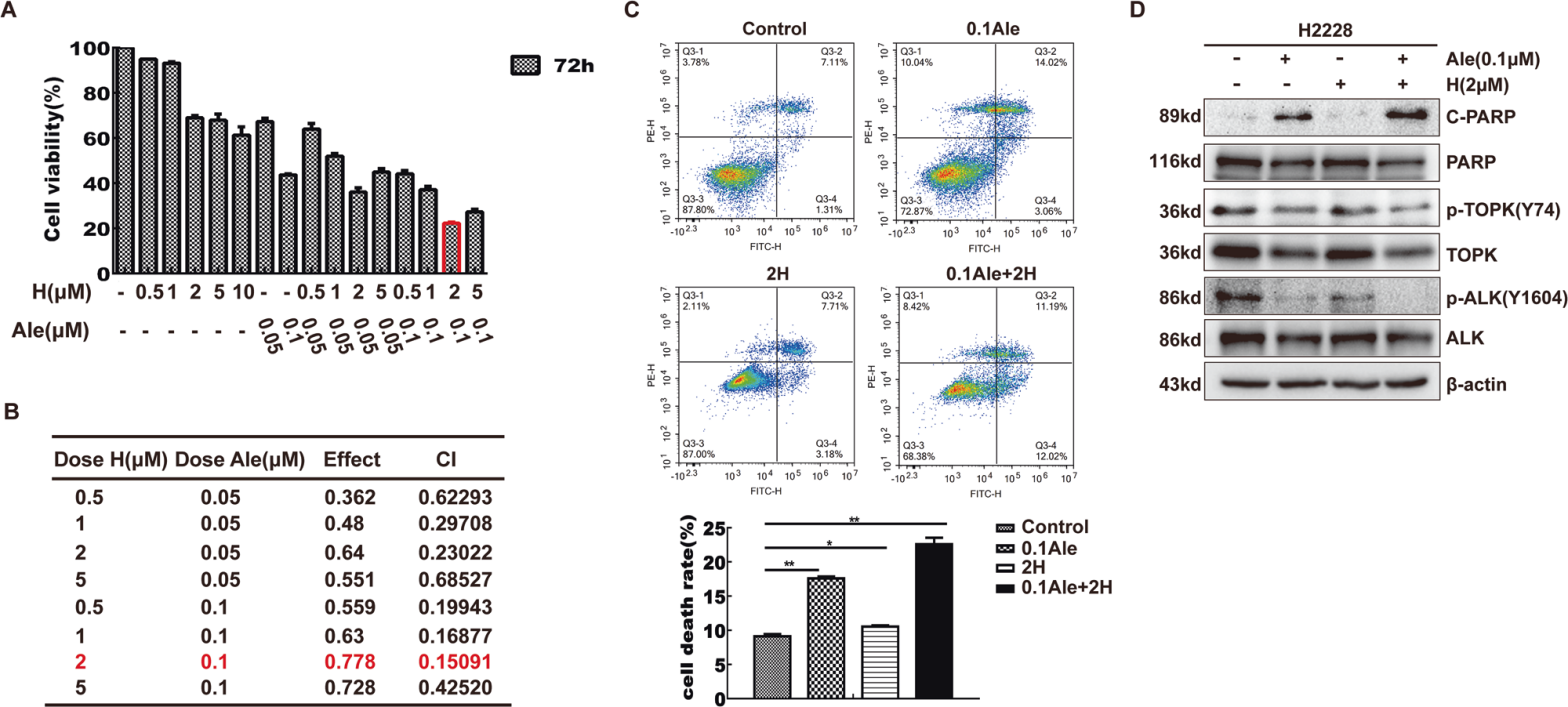
Treatment with a combination of alectinib and HI-032 synergistically inhibited the growth and enhanced the apoptosis of H2228 cells. **A** Alectinib and HI-032 with different concentrations were added to H2228 cells, and the two were used alone or in combination for 72 h, and the cell viability was detected by MTT assay. **B** Combined effects were measured using CI values. Quantification of the potency of the different combinations using the Chou-Talalay method as described in the Materials and Methods section. CI values <1.0 correspond to synergistic inhibition effects. **C** H2228 cells were treated with alectinib (0.1 μΜ) and HI-032 (2 μM) and dead cells were detected by Annexin V-FITC and PI double staining and analyzed by flow cytometry. Data are represented as mean ± standard deviation from triplicate experiments. *P < 0.05, **P < 0.01. **D** H2228 cells were treated with alectinib (0.1 μΜ) and HI-032 (2 μM) for 24 h and the samples were analyzed by western blotting. Data are representative of results from triplicate experiments.

As the inhibition of TOPK could induce the apoptosis of H2228 cells and enhance H2228 cell sensitivity to alectinib, we explored the effect of the combination of HI-032 [21] (TOPK inhibitor) and alectinib (ALK inhibitor) in the following experiments. First, we used different concentrations of alectinib and HI-032 alone or in combination and measured cell proliferation at 72 h by MTT assay. The combination of 0.1 μM alectinib and 2 μM HI-032 resulted in significantly higher inhibition of cell growth relative to other double combinations or single-drug treatments (Fig. 6A). Then, the data were analyzed using the Chou-Talalay method. A CI value <1 indicates a synergistic effect. The experimental data showed that the CI values of these two drugs were all less than 1, indicating that the two drugs had a synergistic effect. The CI value of 0.1 μM alectinib and 2 μM HI-032 was 0.15091, the lowest (Fig. 6B). Subsequently, H2228 cells were treated with 0.1 μM alectinib and 2 μM HI-032 alone or in combination for 24 h and analyzed by flow cytometry. The results indicated that the combination of alectinib and HI-032 promoted cell death (Fig. 6C). Similarly, western blotting analysis showed that the level of cleaved PARP was increased after treatment with the combination of the two drugs, suggesting that the combination of the two drugs promoted the apoptosis of H2228 cells (Fig. 6D and Supplementary S1).

### Treatment with the combination of alectinib and HI-032 suppressed tumor growth in vivo

**Fig. 7:**
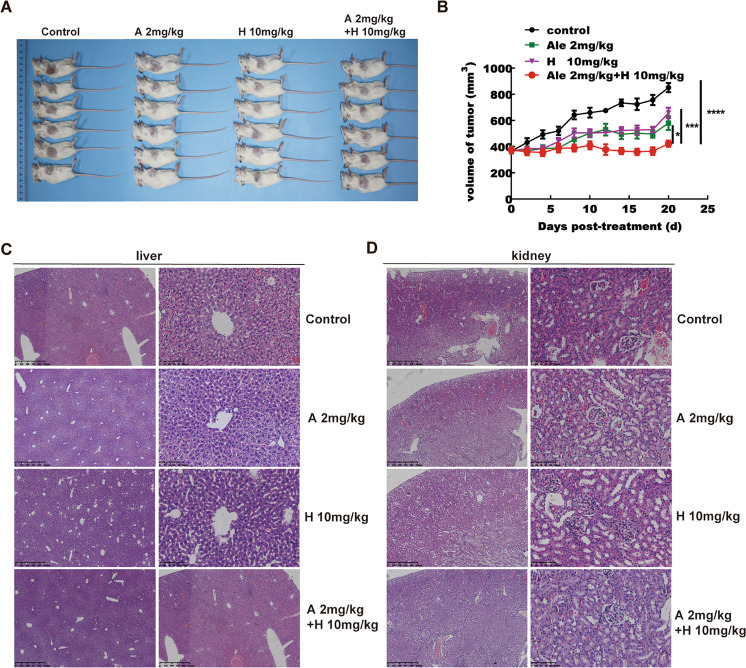
Treatment with the combination of alectinib and HI-032 suppressed tumor growth in vivo. **A**, **B** Mice were treated by intraperitoneal injection with vehicle, alectinib, HI-032 or alectinib plus HI-032 for 20 days. Significant differences were determined by factorial analysis of variance. *P < 0.05, ***P < 0.001. **C**, **D** HE staining of liver and kidney among the four groups of animal study. Representative images were shown. Magnifications, ×40, ×200, scale bar = 100 µm.

The above results indicated that the combination of the two drugs could effectively inhibit cell growth and induce apoptosis ex vivo, and these effects urgently needed to be confirmed in vivo. Thus, we performed an animal study to investigate the effectiveness of the combination of alectinib and HI-032 in preventing tumor growth of H2228 cells. Animals were randomly divided into four groups: the alectinib (2 mg/kg) group, HI-032 (10 mg/kg) group, combined group and control group. The details are described in the methods section. The results revealed that the combination of alectinib and HI-032 effectively inhibited tumor growth in vivo compared with the negative control treatment or the single-drug treatment (Fig. 7A, B), and there was no significant difference in body weight and the morphology of liver and kidney among the four groups (Supplementary S4 and Fig. 7C, D). In all, the combination of alectinib and HI-032 also suppressed tumor growth in vivo.

## Discussion

Many studies have shown that ALK is a specific target for cancer therapy, and ALK inhibitors have been approved for use in the treatment of patients with advanced NSCLC who are confirmed to have *ALK* arrangements [22]. However, the emerging problem of drug resistance prompted us to further explore the mechanism by which ALK participates in oncogenesis. In our study, TOPK was phosphorylated on tyrosine residue Y74 by ALK and promoted tumor growth of ALK-positive lung cancer cells. Additionally, the TMT data predicted some potential downstream molecules that are affected by this phosphorylation modification, and the prediction was proven to be reliable. This novel ALK-TOPK pathway may also function in other ALK-positive cancers, such as diffuse large B-cell lymphoma or inflammatory breast cancer [23], and TOPK could be a favorable target for the treatment of ALK-positive cancers.

Compared with other molecules directly downstream of ALK, such as Smad4, STAT3, PI3K and PLC-γ, TOPK is a better target for cancer therapy. Many clinical studies have shown that TOPK is a predicted marker of poor prognosis in patients with a variety of tumors, such as colorectal cancer [24], gastric carcinoma [25], and ovarian cancer [26]. TOPK might be an independent predictor for overall survival (OS) in human patients with cancers [27]. Several inhibitors such as HI-032 and SKLB-C05 have been identified to inhibit TOPK activity and its downstream pathways by targeting TOPK [9]. Moreover, TOPK is actually a cancer/testis antigen (CTA) with highly specific expression in the testis and various tumors [10]. CTAs are priority targets for tumor immunotherapies because of their immunogenicity and tumor specificity [28], and recombinant tumor vaccines, such as MAGE-A3 antigen, have been used in clinical trials of lung cancer treatment [29]. Our study showed that TOPK was highly expressed in ALK-positive NSCLC. Thus, cancer vaccine–mediated immunotherapy and/or inhibition of TOPK kinase function could be beneficial in the treatment of ALK-positive NSCLC.

Similar to other TKIs, the clinical use of ALK inhibitors is almost universally limited by drug resistance. The drug combination treatment strategy is one method for overcoming or delaying drug resistance, and selecting drug targets from the same or bypass pathways is supported [30]. It has been reported that combining crizotinib with everolimus synergistically inhibits the proliferation of ALK-positive anaplastic large cell lymphoma and lung cancer cells [31]. In addition, apatinib restores sensitivity to alectinib by increasing cell apoptosis and inhibiting cell viability [32]. Moreover, Bivona et al. demonstrated that upfront coinhibition of ALK-MEK enhances the initial response and delays acquired resistance in ALK-positive adenocarcinoma [33]. Here, the combination of a TOPK inhibitor and alectinib synergistically promoted cell apoptosis and hindered cell proliferation ex vivo and in vivo. TOPK has been identified as an oncogenic MEK [11]. It would be more effective to inhibit two oncogenic targets in the treatment of cancer. Therefore, our study provides a promising combination for early treatment to delay drug resistance.

Overall, our findings expand the oncogenic signaling network of ALK, and coinhibition of ALK and TOPK may offer a novel strategy for the treatment of ALK-positive NSCLC patients to delay drug resistance. In the future, the downstream pathways related cell proliferation, apoptosis or kinases of TOPK would be worthy of further study, and the clinical value of TOPK in ALK-positive NSCLC must be further clarified.

## Supplementary information

supplementary 1. The full membrane blots

supplementary 2. MS information

Supplementary 3.Functional enrichment-based clustering analysis of the KEGG pathway involved in ALK-TOPK signaling

Supplementary 4. the body weight changes of mice

supplementary legends

checklist

## Data Availability

All data generated or analyzed during this study are available from the corresponding author on reasonable request.
