# Supplementary material for: Inhibiting ALK-TOPK signaling pathway promotes cell apoptosis of ALK-positive NSCLC
Source: Cell Death Dis. 2022 Sep 27;13(9):828. doi: 10.1038/s41419-022-05260-3 (PMC9515217; doi:10.1038/s41419-022-05260-3)

Figure 1A

p-Tyrosine

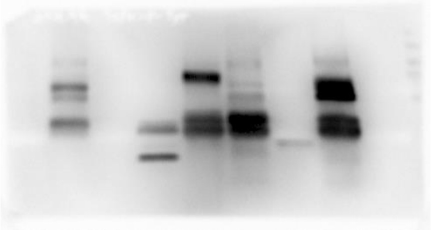

Figure 1D

p-TOPK(Y74)

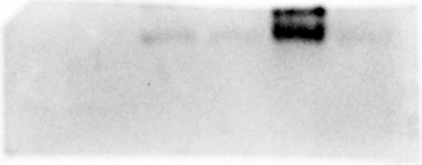

ALK

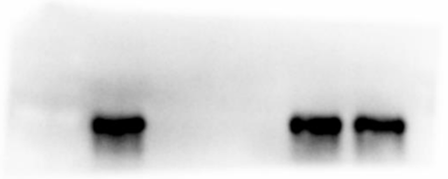

His-TOPK

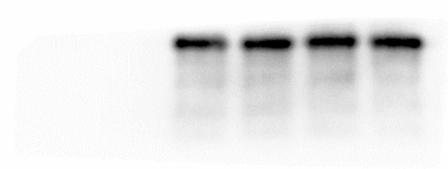

Figure 3A

ALK

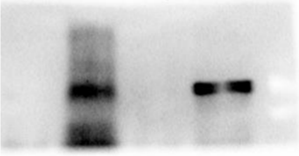

TOPK

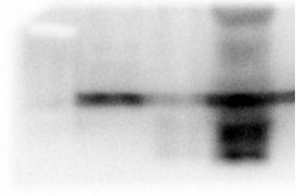

Figure 3B left panel

p-TOPK(Y74)

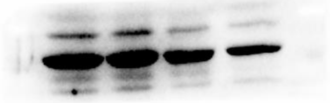

TOPK

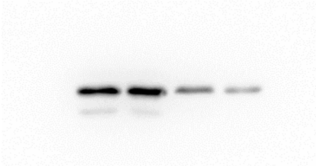

p-ALK(Y1604)

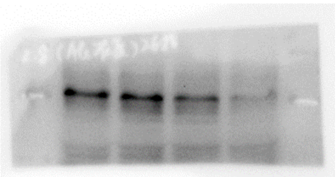

ALK

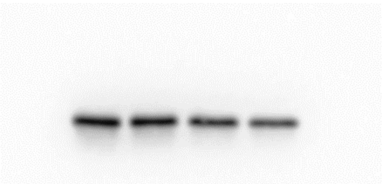

β-actin

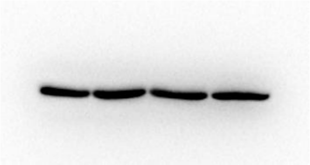

Figure 3B right panel

p-TOPK(Y74)

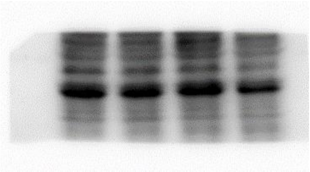

TOPK

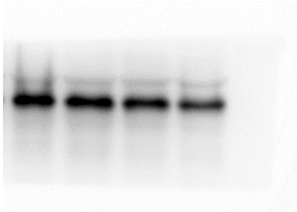

p-ALK(Y1604)

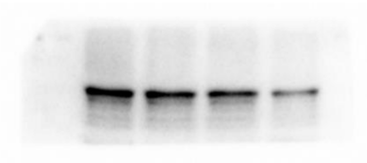

ALK

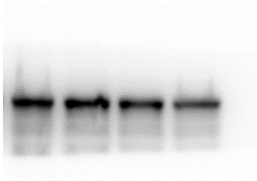

β-actin

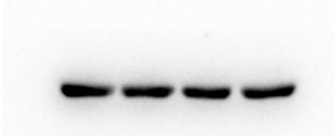

Figure 3C left panel

ALK

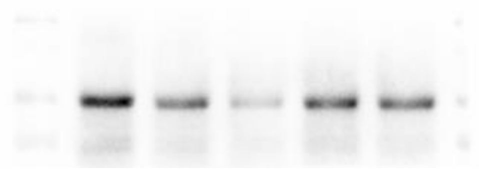

β-actin

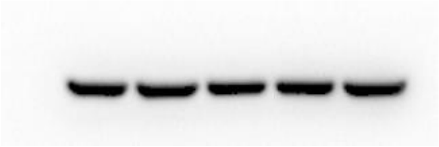

Figure 3C right panel

p-TOPK(Y74)

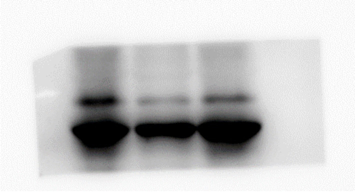

TOPK

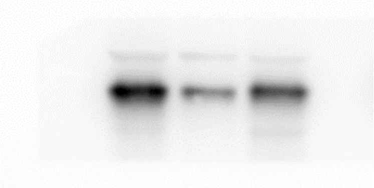

p-ALK(Y1604)

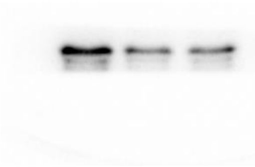

ALK

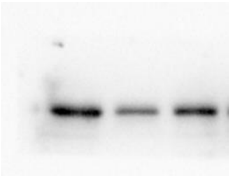

β-actin

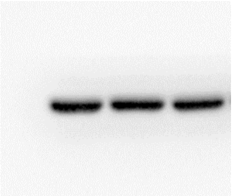

Figure 3D left panel

p-TOPK(Y74)

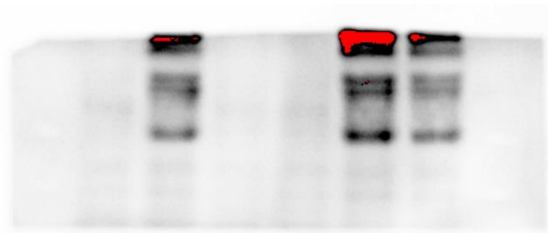

HA

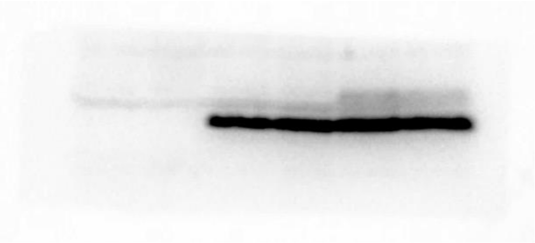

p-ALK(Y1604)

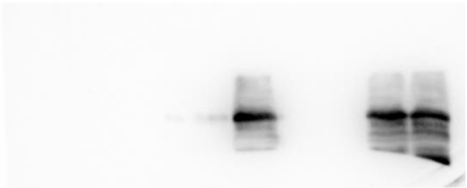

flag

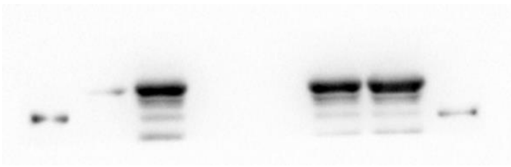

β-actin

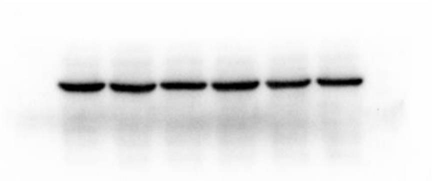

Figure 3D right panel

p-TOPK(Y74)

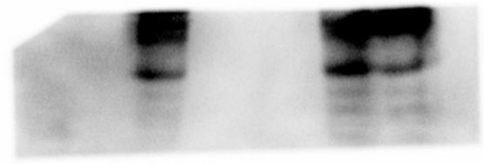

HA

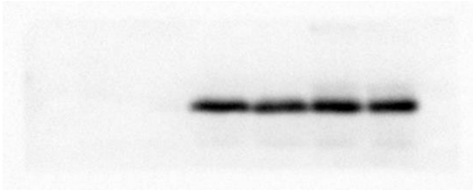

p-ALK(Y1604)

flag

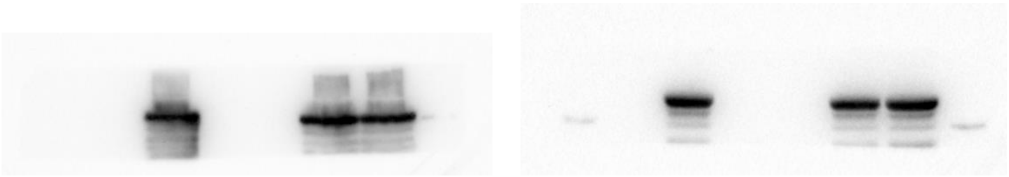

β-actin

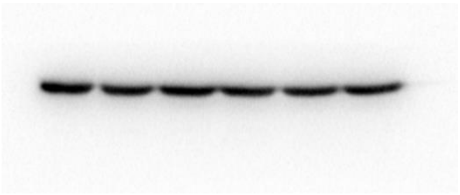

Figure 3E

ALK

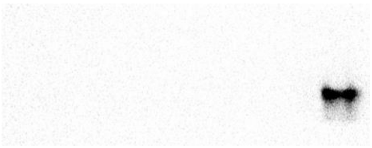

TOPK

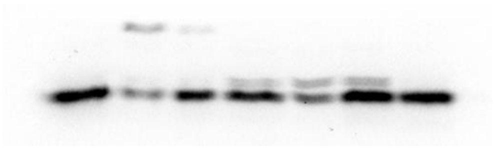

actin

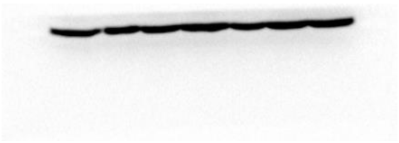

Figure 3F left panel

p-TOPK(Y74)

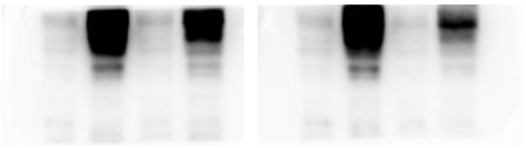

TOPK

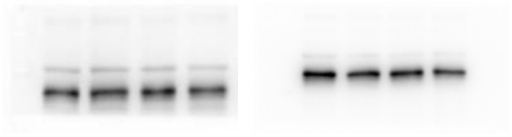

p-ALK(Y1604)

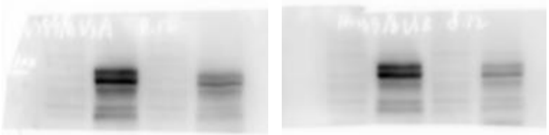

ALK

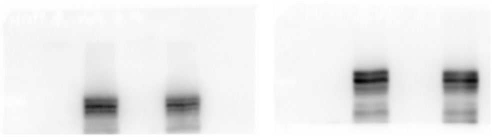

β-actin

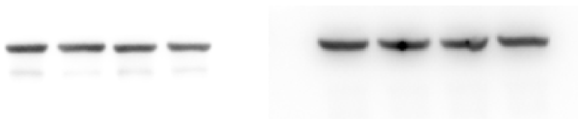

Figure 3F right panel

p-TOPK(Y74)

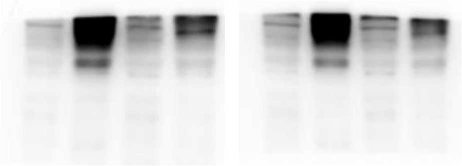

TOPK

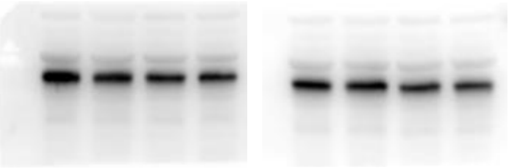

p-ALK(Y1604)

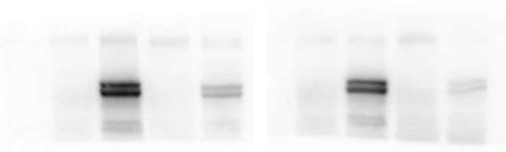

ALK

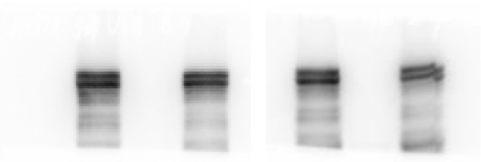

β-actin

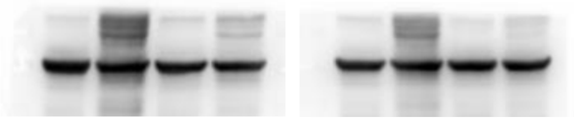

Figure 3G

TOPK

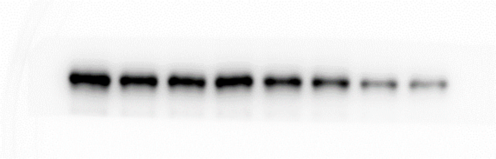

Tubulin

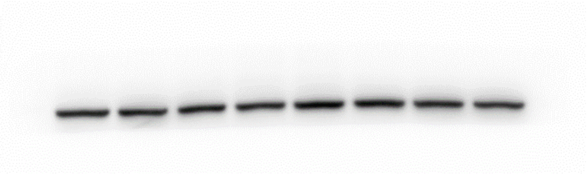

Figure 4A

TOPK

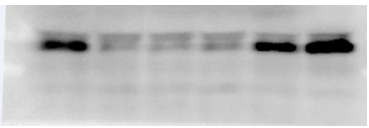

β-actin

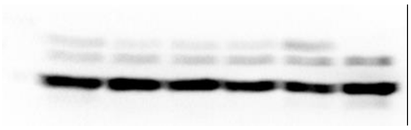

Figure 4C

C-PARP

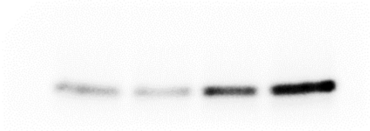

PARP

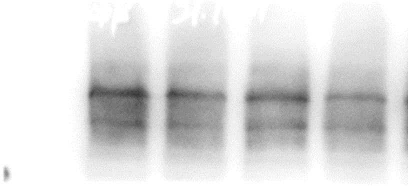

TOPK

β-actin

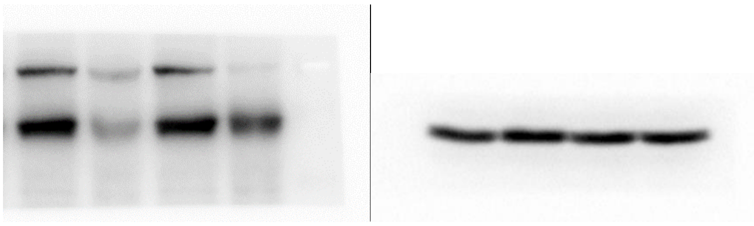

Figure 5D

p-JNK(T183/Y185)

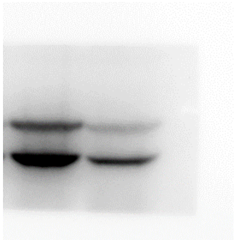

JNK2

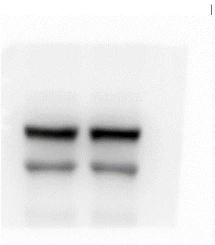

p-ATF2(T71)

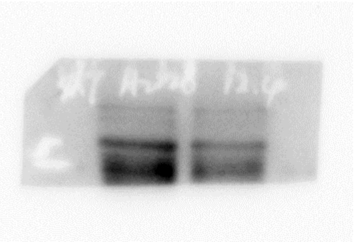

ATF2

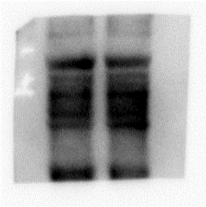

TOPK

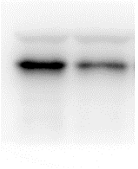

β-actin

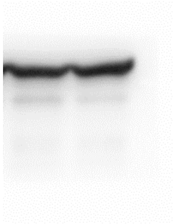

Figure 6D

C-PARP

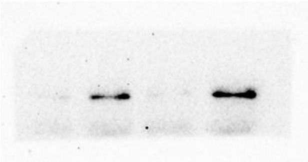

PARP

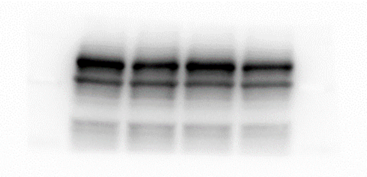

p-TOPK(Y74)

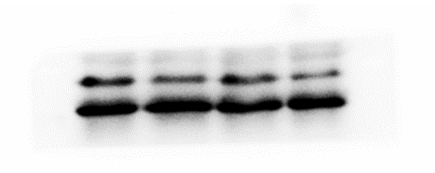

TOPK

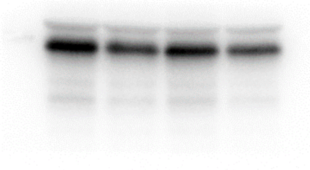

p-ALK(Y1604)

ALK

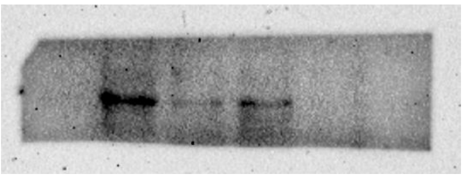

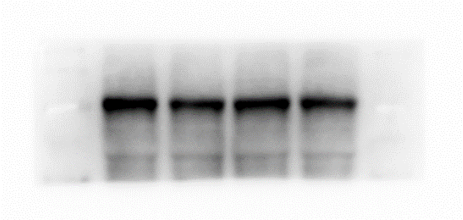

β-actin

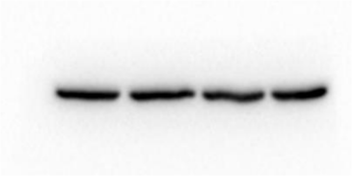

Supplement: Supplementary file 1 — supplementary 1. The full membrane blots [file 41419_2022_5260_MOESM1_ESM.pdf]
