# Supplementary figures and images for: Inhibiting ALK-TOPK signaling pathway promotes cell apoptosis of ALK-positive NSCLC
Source: Cell Death Dis. 2022 Sep 27;13(9):828. doi: 10.1038/s41419-022-05260-3 (PMC9515217; doi:10.1038/s41419-022-05260-3)

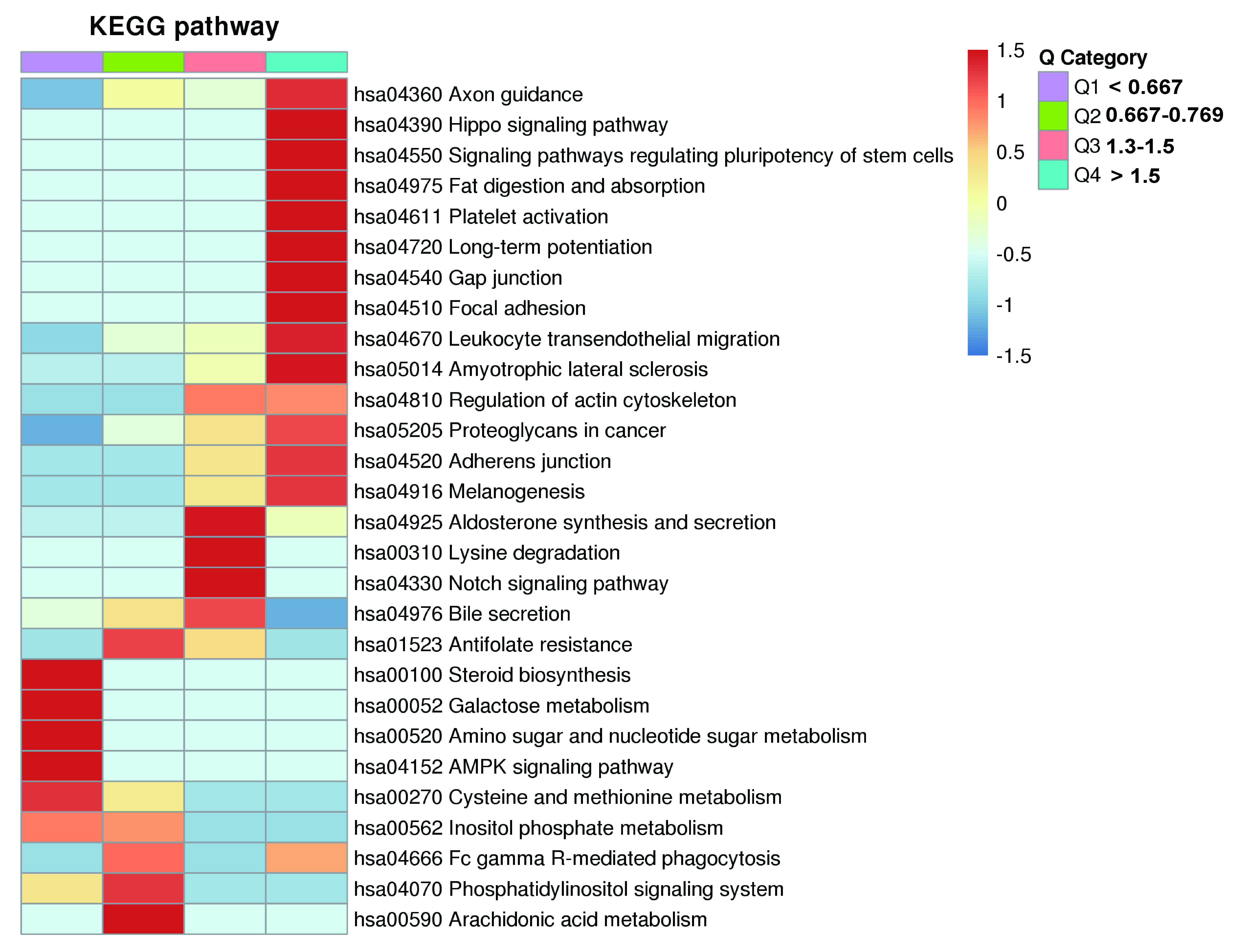

Supplement: Supplementary file 3 — Supplementary 3.Functional enrichment-based clustering analysis of the KEGG pathway involved in ALK-TOPK signaling [file 41419_2022_5260_MOESM3_ESM.tif]
