# Supplementary material for: Inhibiting ALK-TOPK signaling pathway promotes cell apoptosis of ALK-positive NSCLC
Source: Cell Death Dis. 2022 Sep 27;13(9):828. doi: 10.1038/s41419-022-05260-3 (PMC9515217; doi:10.1038/s41419-022-05260-3)
Supplement: Supplementary file 5 — supplementary legends [file 41419_2022_5260_MOESM5_ESM.docx]

**Supplementary. S2** A total of 5091 phosphosites in 2450 proteins were identified and quantified.

**Supplementary. S3** Functional enrichment-based clustering analysis of the KEGG pathway involved in ALK-TOPK signaling.

**Supplementary. S4** In animal study, the body weight changes of mice were monitored every 2 days, and the curve was plotted.
